# Four-dimensional flow magnetic resonance assessment of alcohol septal ablation for hypertrophic obstructive cardiomyopathy and surgical valve replacement for aortic valve stenosis

**DOI:** 10.3389/fcvm.2025.1529350

**Published:** 2025-04-08

**Authors:** Rie Aoyama, Shinichi Okino, Shigeru Fukuzawa

**Affiliations:** Department of Cardiology, Heart and Vascular Institute, Funabashi Municipal Medical Center, Chiba, Japan

**Keywords:** 4D flow MRI, hypertrophic cardiomyopathy, aortic valve stenosis, alcohol septal ablation, aortic valve replacement

## Abstract

**Background:**

Hypertrophic cardiomyopathy sometimes complicates left ventricular (LV) outflow tract obstruction. Alcohol septal ablation (ASA) is indicated for drug-refractory hypertrophic obstructive cardiomyopathy (HOCM). Moreover, with an aging population, aortic valve stenosis (AS) is increasing, and surgical aortic valve replacement (SAVR) is indicated in these cases. Both AS and HOCM have stenosis at the exit of the LV and there is a difference in valvular and/or muscular stenosis. However, it is not clear how the release of stenosis affects blood flow. We investigate the influence of ASA and SAVR on blood flow using four-dimensional flow phase-contrast magnetic resonance imaging (4D flow MRI).

**Methods:**

In this single-center retrospective observational study, we evaluated the blood flow of eight patients (five patients with HOCM and three patients with AS) before and after the intervention using 4D flow MRI.

**Results:**

The LV-aortic pressure gradient (PG) significantly improved from 79.4 ± 3.9 to 23.0 ± 2.0 mmHg (*p* < 0.001) by SAVR in the patients with AS. However, turbulent kinetic energy value (TKE) loss was not improved. However, the intra-LV PG in patients with HOCM improved from 79.0 ± 54.2 to 8.7 ± 4.0 mmHg (*p* < 0.05) by ASA. TKE loss improved from 7.0 ± 2.0 to 5.0 ± 0.1 mW (*p* < 0.05) and New York Heart Association functional class significantly improved from 2.2 ± 0.5 to 1.1 ± 0.3 (*p* < 0.001) by ASA.

**Conclusions:**

The release of valvular or muscular stenosis has different effects on intra-LV blood flow. ASA reduced TKE loss and 4D flow MRI is useful to evaluate the efficacy of therapeutic interventions.

## Introduction

Hypertrophic cardiomyopathy (HCM) is characterized by asymmetric left ventricular (LV) hypertrophy in the absence of other cardiac or systemic diseases that may cause cardiac hypertrophy (1). Although many patients stay asymptomatic and experience no serious cardiac event during their lifetime, some experience sudden cardiac death, refractory heart failure, repetitive syncope, and/or severe angina. These various clinical presentations are closely associated with narrowing LV capacity and intra-LV obstruction. Septal reduction therapy such as septal myectomy and alcohol septal ablation (ASA) is indicated to relieve symptoms and improve exercise capacity in patients with drug-refractory HCM who have left ventricular outflow tract (LVOT) obstruction (LVOTO) (2, 3). ASA is an optional treatment for patients with symptomatic hypertrophic obstructive cardiomyopathy (HOCM) who are refractory to medical therapy and have high surgery risk due to serious comorbidities or advanced age (4). Increasing evidence has supported the beneficial effect of ASA in improving symptoms associated with HOCM. It has been reported that the improvement of LVOTO by ASA also improves LV myocardial metabolism (5) and LV blood flow (6).

With an aging population, the incidence of valvular heart disease has been increasing, with most cases presenting as aortic valve stenosis (AS) and are increasingly observed to be of a degenerative etiology. Surgical aortic valve replacement (SAVR) is indicated for survival benefit, improvement in symptoms, and improvement in LV systolic function in patients with severe symptomatic AS (7). Outcomes after surgical AVR are excellent in patients who do not have a high procedural risk.

Non-invasive imaging techniques, such as transthoracic echocardiography (TTE) and two-dimensional cine phase-contrast magnetic resonance imaging (MRI), are the standard methods of cardiovascular investigation. In addition to the morphological evaluation by these investigations, blood flow also plays an important role in the assessment of cardiovascular disease. In recent years, four-dimensional (4D) flow MRI has been increasingly applied in the cardiovascular field and enables comprehensive and non-invasive hemodynamic flow assessments. Several reports (6, 8) have demonstrated elevated intra-cardiac energy loss in various cardiovascular diseases.

Blood flow in the LV forms an appropriate vortex flow, which maintains momentum without stopping blood flow in the LV, minimizing energy loss and smoothing the change of direction from the mitral to the aortic valve. However, when the LV is disrupted or flow separation occurs due to AS or HOCM, energy loss increases. This increased energy loss is compensated by an increased workload on the LV. The presence of a stenosis increases the blood flow velocity and increases the dynamic pressure (kinetic energy) while decreasing the static pressure. Then, when the blood passes through the stenotic site, flow separation and turbulence occur, resulting in energy loss, and the static pressure drops without returning to the original pressure. It has been reported that a large energy loss is associated with a poor prognosis (9). Viscous energy loss in blood flow due to turbulent kinetic energy (TKE) generation occurs within the LV.

Both AS and HOCM have stenosis at the exit of the LV, although there is a difference in valvular and/or muscular stenosis, and the release of the stenosis improves subjective symptoms and heart failure. However, it is not clear how the release of stenosis affects intra-LV or LV-aortic blood flow. This study aimed to investigate the effect of ASA and SAVR on intra-LV or LV-aortic blood flow and energy loss in the LV and aortic root as measured by 4D flow MRI in patients with HOCM and LVOTO and patients with severe AS.

## Methods

### Study design and participant recruitment

This study was conducted following the principles of the Declaration of Helsinki and its later amendments. In this single-center retrospective observational study, between January 2018 and February 2021, medical and family history was obtained, along with current drug therapy and assessment of heart failure symptoms using the New York Heart Association (NYHA) classification.

NYHA classification, echocardiographic data, serum brain natriuretic peptide (BNP) level, and LV or LV-intra-aortic blood flow were evaluated before and after the intervention in eight patients (five patients with HOCM and three patients with AS) using 4D flow MRI. Post-procedural data, except for MRI, were obtained after 3 months of treatment and MRI was obtained within 6 months.

### Transthoracic echocardiogram study

We performed two-dimensional, M-mode, and Doppler TTE studies before and after ASA or SAVR using PHILIPS IE 33 (Philips Healthcare, Best, The Netherlands) or GE Vivid E 9 ultrasound systems (GE Healthcare, Boston, MA, USA). TTE was performed preoperatively within a week before surgery and postoperatively approximately 30 days after surgery. We measured the LV dimensions in both diastole and systole, the left atrium (LA) dimensions, and the thickness of the intraventricular septum (IVS) and LV posterior wall (LVPW) using M-mode. We also measured the LV ejection fraction (LVEF) using a modified Simpson's method. Peak velocity in the aortic valve or the LVOT and pressure gradient (PG) between the aorta and LV were calculated using LVOT Doppler waveforms, LVOT diameter, heart rate, and the continuity equation as per standard techniques. We also measured LV diastolic function [the E/A (peak early transmitral filling velocity/peak late transmitral filling velocity) and the E/e' (peak early transmitral filling velocity/peak early diastolic mitral annulus velocity) of the septal side or lateral side on tissue Doppler imaging].

### ASA procedure for patients with HOCM and LVOTO

We performed ASA and assessed serial MRI in five patients with HOCM. Indication for HOCM was determined according to the following criteria: (1) symptoms were life-limiting after optimization of medication, (2) resting or provoked gradient >50 mmHg that was confirmed by at least one method during simultaneous pressure recordings, and (3) appropriate target branch(es) leading to the septal myocardium were responsible for intra-LV obstruction. ASA was performed as previously described (4, 10).

### SAVR procedure for patients with AS

AS was diagnosed by TTE and all the patients had clinical symptoms such as shortness of breath and/or chest pain. We performed SAVR with a bioprosthetic valve (INSPIRIS RESILIA 19 mm, Edwards Lifesciences, Irvine, CA, USA) and assessed serial MRI in three symptomatic patients with severe AS.

### Cardiac MRI and 4D flow MRI examination

All patients underwent cardiac MRI before and after ASA or SAVR, along with 4D flow MRI, on a 1.5T MRI system (MR SIGNA HDxt, GE Healthcare, Waukesha, WI, USA), with a maximum gradient strength of 33 mT/m and a maximum slew rate of 120 T/m/s. The postoperative MRI was performed within 6 months, and the average time interval for all cases was 5.6 months after the procedure. The standard-of-care cardiac MRI included electrocardiogram-gated steady-state free procession cine cardiac MRI and late gadolinium enhancement (LGE) if renal function was preserved (11). A commercially available eight-channel phased array body coil was applied. For the clinical purposes of assessment of chamber size and extent of scarring, two-dimensional (2D) fast imaging employing steady-state acquisition (FIESTA) based on a steady-state free precession sequence for cine images was conducted and an inversion recovery prepared fast gradient echo sequence for LGE images was acquired in the short, vertical long-axis, and horizontal long-axis orientations with a slice thickness gap of 9 mm/0 mm.

The LV end-diastolic and LV end-systolic volume indexes (LVEDVI and LVESVI), LV stroke volume index (LVSVI) and LVEF, and LV mass index (LVMI) were quantified using short-axis images from 2D FIESTA. The field of view was adjusted for each subject to fully encompass the heart.

### Post-processing and quantitative analysis of 4D flow MRI

Time-resolved two-dimensional phase-contrast MRI of the three-chamber plane with three-directional velocity encoding was implemented during breath-holding with prospective electrocardiogram gating. The imaging parameters for 4D flow MRI were as follows: voxel size: 6.000 mm^3^ × 1.5625 mm^3^ × 1.5625 mm^3^, repetition time (TR): 2.955–9.932 ms, echo time (TE): 1.232–4.516 ms, flip angle (FA): 20°–60°, number of excitations (NEX): 0.75–1.9, and view per cardiac phase 12–20. Post-processing and quantitative analysis of 4D flow MRI were performed using commercially available post-processing software (iT Flow version 1.8.7; Cardio Flow Design Inc., Japan). We described the region of interest (ROI) as the area surrounded by the LV endocardium, sinotubular junction, and mitral annulus within the three-chamber plane. The ROI was traced manually in each single phase of the cine MRI, allowing the software to automatically calculate the viscous energy loss (mW/m) within the region in each phase based on a previously established formula (8, 12). Intra-cardiac energy loss (EL) can be calculated as follows:

$$\mathrm{Energy}\;\mathrm{loss}\;=\int \mu \sum_{ı\;j}\;{\left(\frac{\partial \mu j}{\partial \chi ı}+\frac{\partial \mu ı}{\partial \chi j}\right)}^{2}dv$$

where *µ* represents blood viscosity, *i* and *j* are arbitrary directions on the Cartesian axes, and *dv* is the volume increment of the segmented lumen. Energy loss inside the LV and energy loss from the LV to the ascending thoracic aorta during one cardiac cycle were calculated from the extracted lumen of the LV and from the LV to the ascending aorta, respectively. Artifacts caused by the bioprosthetic valve were removed to calculate the actual energy loss because they disturb energy loss. This energy loss was integrated by one cardiac cycle, and TKE loss was calculated backward from this energy loss.

### Statistical methods

Continuous variables are presented as means ± standard deviations. Categorical variables are presented as numbers (percentages). All statistical analyses were performed using IBM SPSS Statistics software (version 21 for Windows, SPSS Inc., Chicago, IL, USA). Both continuous and categorical variables were compared between pre-ASA/SAVR and post-ASA/SAVR using paired tests (Wilcoxon signed rank and McNemar tests). Statistical significance was defined as a *p*-value of <0.05.

## Results

### Participants and baseline characteristics

4D flow MRI parameters were compared between patients with HOCM (*n* = 5) or AS (*n* = 3) before and after ASA or SAVR from between January 2018 and February 2021.

The baseline characteristics of the patients enrolled in our study are listed in Table 1. The average ages of the patients with HOCM and AS were 76.6 ± 6.1 and 75.7 ± 8.1 years, respectively. Comorbidities such as hypertension, dyslipidemia, diabetes mellitus, and smoking history were not statistically significantly different between the groups.

**Table 1:** Baseline characteristics of the patients with HOCM and AS.

Baseline characteristics	HOCM (*n* = 5)	AS (*n* = 3)	*p*
Age (years)	76.6 ± 6.1	75.7 ± 8.1	0.87
Male, *n* (%)	1 (20)	1 (33.3)	0.67
BMI, kg/m^2^	22.3 ± 4.5	22.9 ± 1.6	0.81
BSA, m^2^	1.46 ± 0.18	1.45 ± 0.11	0.91
NYHA functional class	2.2 ± 0.5	2.7 ± 0.6	0.31
Syncope, *n* (%)	2 (40)	0 (0)	0.67
Family history of SCD, *n* (%)	0 (0)	—	—
Family history of HCM, *n* (%)	1 (20)	—	—
Hypertension, *n* (%)	3 (60)	3 (100)	0.67
Dyslipidemia, *n* (%)	1 (20)	3 (100)	0.33
Diabetes mellitus, *n* (%)	1 (20)	1 (33.3)	0.67
Past or current smoking, *n* (%)	1 (20)	1 (33.3)	0.67
Coronary artery disease, *n* (%)	0 (0)	1 (33.3)	0.07
Atrial fibrillation, *n* (%)	0 (0)	0 (0)	—
ICD or pacemaker implantation	1 (20)	0 (0)	0.78
Medication			
β blockers, *n* (%)	5 (100)	2 (66.7)	0.78
Calcium channel antagonists, *n* (%)	4 (80)	2 (66.7)	0.67
Na channel blockers, *n* (%)	3 (60)	0 (0)	0.35

HOCM, hypertrophic obstructive cardiomyopathy; AS, aortic valve stenosis; BMI, body mass index; BSA, body surface area; NYHA, New York Heart Association; SCD, sudden cardiac death; HCM, hypertrophic cardiomyopathy; ICD, implantable cardioverter defibrillator.

Data are expressed as mean ± standard deviation or number of the patients (percentage). *p*-value compares for continuous variables using student's *t*-test and categorical and ordinal variables using chi-square test.

### The change of PG and TKE loss in AS before and after SAVR

**Figure 1:**
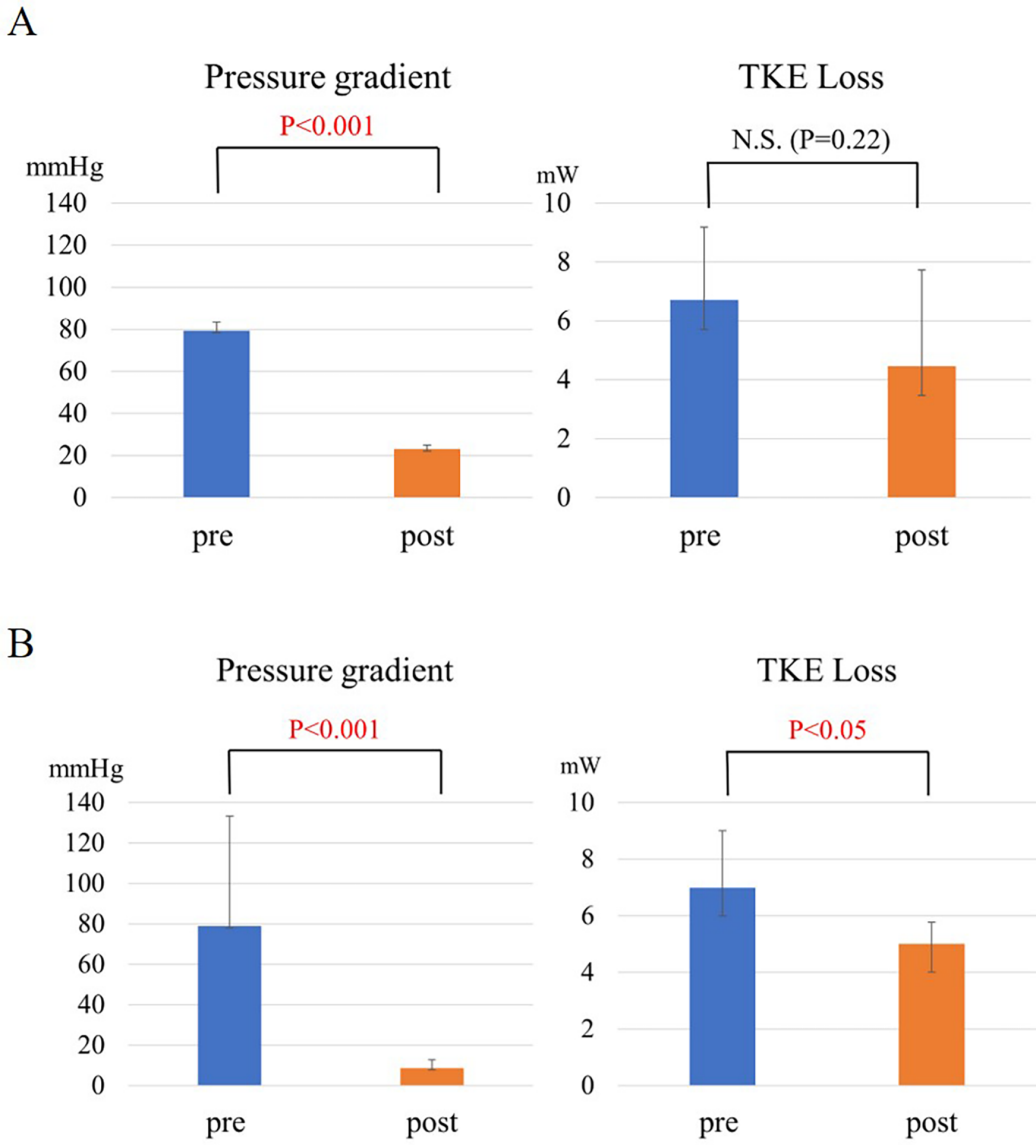
(**A**) The change in pressure gradient and TKE loss in AS before and after SAVR. (**B**) The change in intra-left ventricular pressure gradient and TKE loss in HOCM before and after ASA.

The LV-aortic PG was significantly improved from 79.4 ± 3.9 to 23.0 ± 2.0 mmHg (*p* < 0.001) and the NYHA functional class was also significantly improved from 2.7 ± 0.6 to 1.3 ± 0.6 by SAVR in the patients with severe AS (*p* < 0.001) (Table 2). In contrast, TKE loss in LV was not significantly improved by AVR in the AS group (TKE: from 6.7 ± 2.5 to 4.5 ± 3.3 mW, *p* = 0.22) (Figure 1A).

**Table 2:** Characteristics of AS patients and parameters before and after SAVR and HOCM patients and parameters before and after ASA.

AS patients	Before SAVR	After SAVR	*p*
NYHA functional class	2.7 ± 0.6	1.3 ± 0.6	<0.001
BNP (pg/ml)	259.4 ± 147.5	89.4 ± 56.1	0.07
UCG			
LVEF, %	68.0 ± 3.6	64.8 ± 4.2	0.26
IVS thickness, mm	11.2 ± 0.8	10.7 ± 1.4	0.22
LVPW thickness, mm	11.2 ± 1.0	11.1 ± 2.0	0.44
LA diameter, mm	37.7 ± 9.0	37.8 ± 4.4	0.51
LVDd, mm	38.5 ± 8.5	41.4 ± 2.9	0.74
LVDs, mm	30.2 ± 6.8	26.8 ± 2.4	0.29
AV maximum velocity, m/s	4.4 ± 0.1	2.4 ± 0.1	<0.001
AV maximum PG, mmHg	79.4 ± 3.9	23.0 ± 2.0	<0.001
E/A	0.6 ± 0.2	1.1 ± 0.3	0.96
E/'e (IVS)	14.6 ± 4.3	16.0 ± 2.9	0.69
E/'e (lat)	12.7 ± 3.3	12.0 ± 1.7	0.4
MRI imaging			
LVEDVI	64.3 ± 5.2	48.4 ± 13.9	0.04
LVESVI	23.8 ± 11.3	18.7 ± 9.1	0.28
SVI	40.5 ± 14.2	29.7 ± 5.2	0.12
LVEF (%)	68.0 ± 3.6	64.8 ± 4.2	0.26
the presence of LGE	0 (0)	–	–
HOCM patients	Before ASA	After ASA	*p*
ASA procedure			
the amount of injected alcohol (ml)	3.3 ± 1.6	–	
the number of alcohols injected septal branches	1.8 ± 0.8	–	
peak CK (IU/L)	1327.4 ± 887.1	–	
peak CKMB (IU/L)	201.2 ± 142.0	–	
NYHA functional class	2.2 ± 0.5	1.1 ± 0.3	<0.001
BNP (pg/ml)	440.5 ± 283.1	94.4 ± 54.0	0.02
UCG			
LVEF, %	72.2 ± 4.5	70.9 ± 5.9	0.66
IVS thickness, mm	16.2 ± 2.1	12.2 ± 2.4	0.04
LVPW thickness, mm	12.1 ± 2.9	11.3 ± 0.5	0.55
LA diameter, mm	40.7 ± 2.7	37.5 ± 4.9	0.18
LVDd, mm	39.5 ± 7.2	38.8 ± 3.9	0.85
LVDs, mm	22.0 ± 4.7	23.7 ± 2.5	0.60
LVOT maximum velocity, m/s	4.2 ± 1.8	1.4 ± 0.4	0.02
LVOT maximum PG, mmHg	79.0 ± 54.2	8.7 ± 4.0	0.04
E/A	0.8 ± 0.2	0.7 ± 0.3	0.77
E/'e (IVS)	24.5 ± 7.2	18.2 ± 9.3	0.26
E/'e (lat)	18.4 ± 8.2	15.1 ± 4.9	0.46
SAM, n (%)	5 (100)	0 (0)	<0.001
MRI imaging			
LVEDVI	57.7 ± 11.3	59.7 ± 21.2	0.74
LVESVI	12.9 ± 4.1	13.8 ± 5.0	0.76
SVI	44.7 ± 14.5	45.9 ± 18.4	0.84
LVEF (%)	76.1 ± 11.2	76.3 ± 7.3	0.97
the presence of LGE	2 (40)	–	–

AS, aortic valve stenosis; SAVR, surgical aortic valve replacement; NYHA, New York Heart Association; BNP, brain natriuretic peptide; UCG, ultrasonic cardiography; LVEF, left ventricular ejection fraction; IVS, interventricular septum; LVPW, left ventricular posterior wall; LA, left atrium; LVDd, left ventricular dimension in diastole; LVDs, left ventricular dimension in systole; AV, aortic valve; PG, pressure gradient; E/A, peak early diastolic LV filling velocity/peak atrial filling velocity ratio; E/e’ (IVS) and E/e’ (lat), the ratio between standard Doppler derived transmitral early diastolic velocity (E) and pulsed Doppler derived early diastolic velocity of the mitral annulus (e’) measured at the septal site and at the lateral site of the mitral annulus; LVEDVI, left ventricular end diastolic volume index; LVESVI, left ventricular end systolic volume index; SVI, stroke volume index; LGE, late gadolinium enhancement; HOCM, hypertrophic obstructive cardiomyopathy; ASA, alcohol septal ablation; CK, creatine kinase; CKMB, creatine kinase MB; LVOT, left ventricular outflow tract; SAM, systolic anterior motion of the mitral valve.

Data are expressed as mean ± standard deviation or number of the patients (percentage). P-value compares for continuous variables using Student's t-test.

### The change of PG and TKE loss in HOCM before and after ASA

The intra-LV PG was improved from 79.0 ± 54.2 to 8.7 ± 4.0 mmHg (*p* < 0.05) and SAM in all the patients with HOCM was resolved by ASA (Table 2). TKE loss was significantly improved from 7.0 ± 2.0 to 5.0 ± 0.1 mW (*p* < 0.05) (Figure 1B).

## Case presentations

### Representative case of AS

**Figure 2:**
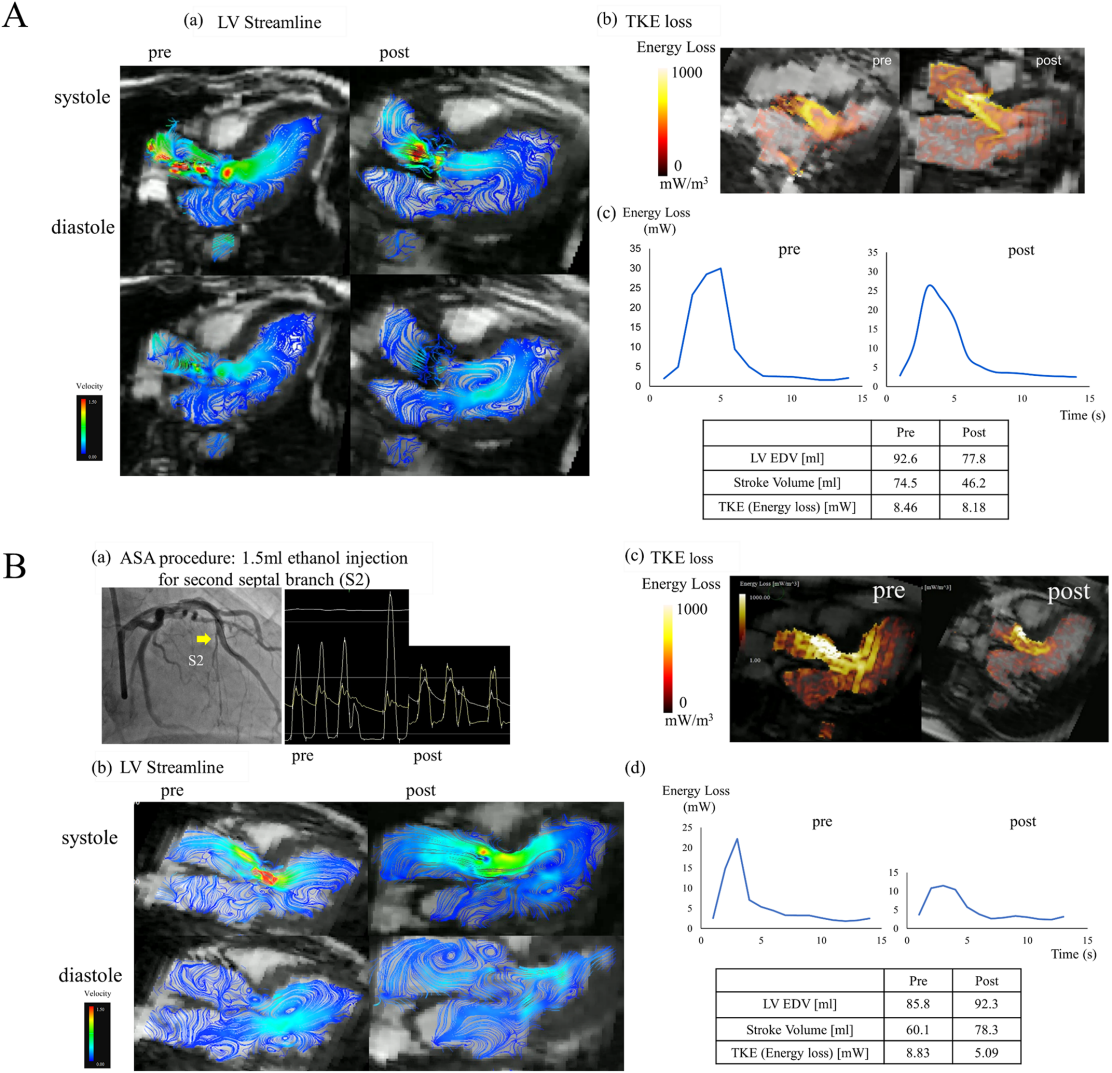
(**A**) A representative case of SAVR. (a) Left ventricular streamline images of 4D flow MRI in the long-axis before and after SAVR. (b) TKE loss in the long-axis before and after SAVR. (c) The graph of viscous energy loss over time before and after SAVR. (**B**) A representative case of ASA. (a) ASA procedure: 1.5 ml ethanol injection in the second septal branch and simultaneous pressure measurements in the aorta and left ventricle before and after ASA. (b) Left ventricular streamline images of 4D flow MRI in the long-axis before and after ASA. (c) TKE loss in the long-axis before and after ASA. (d) The graph of viscous energy loss over time before and after ASA.

A 72-year-old man with anginal symptoms was diagnosed with severe AS by TTE, and the decision was made to perform SAVR. SAVR with a bioprosthetic valve (INSPIRIS RESILIA 19 mm, Edwards Lifesciences, Irvine, CA, USA) was performed; cardiac MRI was performed 3 months after SAVR to compare with preoperative results. LV streamline images of 4D Flow MRI in the long-axis image showed that, unlike HOCM, there were relatively large vortex flows in the LV. Moreover, its position, size, and number in the LV did not change after SAVR (Figure 2A-a). The value of TKE loss did not change (Figure 2A-b). However, its pattern changed and the peak of TKE loss was shifted forward (Figure 2A-c).

### Representative case of HOCM

A 69-year-old woman had been diagnosed with HOCM and LVOTO by TTE and catheterization. She had no history of sudden cardiac death in her family, and her genetic background of HCM was unclear; however, it was a familiar form of HOCM with asymmetrical septal hypertrophy. She had heart failure of NYHA functional classification II and an intra-LV PG of 62.5 mm Hg. Despite administration of a β-blocker (bisoprolol, 5 mg) and a class Ia drug (cibenzoline, 150 mg), her symptoms were not well controlled. Her chest discomfort and dyspnea on exertion gradually worsened with NYHA functional class II with moderate limitation (class IIm). Thus, she had drug-resistant HOCM and ASA was performed. ASA was performed by injecting 1.5 ml of ethanol into the second septal branch. The PG in the LV improved from 62.5 to 19.8 mmHg, and SAM disappeared postoperatively (Figure 2B-a). CK (CKMB) was 661 (81) U/L. Cardiac MRI was performed 3 months after ASA to compare with preoperative results. LV streamline images of 4D flow MRI in the long-axis image showed vortex flows toward the posterior mitral leaflet (PML) and posterior LV wall before ASA, and these vortex flows disappeared after ASA. Besides the vortex flows in the LV, the small vortex flows in the left atrium found before ASA improved after ASA (Figure 2B-b). TKE loss was also improved both in the pattern and value (Figure 2B-c). The graph of energy loss over time shows that the peak after ASA improved markedly, and its integral value, TKE, also improved from 8.83 to 5.09 mW (Figure 2B-d).

### Comparison of 4D flows between patients with HOCM and AS before and after intervention

The peak velocity of blood flow was faster and steeper in AS than in HOCM. A larger number and various forms of vortices were more frequently found in HOCM than in AS, especially in the LV under the mitral valve in HOCM. Intra-LV flows showed differences between valvular and muscular stenosis. In HOCM, a large number and various forms of vortex flows were formed. The release of LVOTO by ASA improved vortex flows both in the LV and LA, and the TKE loss also improved accordingly. The reduction of the LV pressure load resulted in a change in the intra-LV blood flow, especially in HOCM.

## Discussion

In AS or HOCM with stenotic or occlusive lesions that obstruct blood flow, vortex flow formation is seen in the LV, which causes energy loss. The increased energy loss results from changes in blood flow due to the obstruction, and it is assumed that the energy loss results in inefficient blood outflow, increased myocardial workload, and progressive myocardial damage (13, 14).

Studies have evaluated hemodynamics using 4D flow MRI in the ascending aorta in the bicuspid aortic valve (BAV) and reported the occurrence of helical and eccentric flow, increased wall shear stress, and abnormal tissue structure in the dilated aortic wall due to increased wall share stress (14, 15). However, there are few reports on the evaluation of LV blood flow. It was reported that patients with HOCM had higher helical flow grades than patients with hypertrophic non-obstructive cardiomyopathy and helical flow grade was associated with higher helix grade (16). Although disease-specific blood flow patterns using 4D flow MRI in valvular heart disease and cardiomyopathy have been demonstrated and are associated with symptoms along with hemodynamics (15, 17–19), few studies have evaluated hemodynamic changes with therapeutic intervention. TTE has been frequently used to assess the effects of ASA in patients with HOCM (4, 20–22), but cannot evaluate intra-LV flow patterns over time. 4D flow MRI allows any cross-section to be analyzed retrospectively, allowing comparison of blood flow in the same cross-section before and after interventions. 4D flow MRI evaluation has shown that ASA reduces the incidence of vortex and/or helical flow, shortens the duration of vortex and helical flow, and increases the size of each vortex flow through the dispersal of the small vortex flow and the expansion of the large vortex flow. These effects result in increased LV blood flow efficiency and ASA optimizes LV blood flow (6, 23).

In this study, we evaluated LV blood flow before and after interventions for AS and HOCM. The small vortex flow in the LV disappeared and the large vortex flow, which is associated with efficient blood flow, improved. ASA also showed a loss of vortex flow, mainly during diastole, especially the small vortex flow around the PML. Furthermore, SAM disappeared and TKE loss was significantly improved. The reasons for these results are as follows. The LV cavity is small in HOCM, and LV inflow and subsequent vortex formation may be altered by ASA-induced LV cavity enlargement, leading to the restoration of diastolic function and reduction of LV and LA pressure. In contrast, AS had fewer small vortexes even before SAVR and TKE loss was not observed after the release of stenosis by SAVR.

Since effective PG reduction was obtained in both groups, it is likely that valvular and muscular stenosis of the LV outflow tract have different effects on LV blood flow and behave differently after its release. Muscular stenosis has a longer-lasting stenotic lesion than valvular stenosis, and the effect on the LV may be different even with the same PG.

Energy loss is calculated from the velocity of each in-plane pixel in 4D flow MRI and reflects the energy loss corresponding to changes in total pressure, including both static and dynamic pressure. Conversely, the PG in catheterization measures only the static PG. This may theoretically explain the discrepancy between improved energy loss and improved PG, as well as the differential impact on LV blood flow due to muscular and valvular stenosis. This study evaluated the blood flow and energy status within the LV, which could not be expressed by PG alone.

This study demonstrated that ASA reduced energy loss within the LV, which was detected by serial 4D flow MRI. It is still debated whether to use turbulent flow or laminar flow to estimate energy loss. Energy loss was estimated using turbulent flow in this study. Such quantification appears to be a promising approach for assessing cardiac workload and a promising method for following the results of an intervention with a timeline. 4D flow cardiac MRI was considered an appropriate candidate modality for continuous assessment of therapeutic interventions for diseases with LVOT or AV stenosis.

### Limitations

Our study has several limitations. First, it is a single-center, retrospective study. The study includes the small number of patients enrolled due to the limited capacity for patient selection at our hospital. Second, although the post-procedure evaluations were performed after a certain period, it is undeniable that the different levels of invasiveness of the procedures (open heart surgery for AS and catheter intervention for HOCM) may have influenced the results. Third, especially in SAVR for AS, we used the same type and size of bioprosthetic valve, but the bioprosthetic valve might have an impact on the evaluation of blood flow on MRI. Finally, the reconstructed voxel resolution of the 4D flow MRI was anisotropic voxels, which may have affected the accuracy of the results. Finally, tracing the region of interest in each phase of the cardiac cycle was done manually and was time-consuming to analyze.

## Conclusions

The release of valvular or muscular stenosis shows different effects on intra-LV blood flow. ASA reduced TKE loss within the LV, as detected by serial 4D flow MRI. 4D flow MRI is useful for evaluating the hemodynamics and the efficacy of therapeutic interventions.

## Data Availability

The original contributions presented in the study are included in the article/Supplementary Material, further inquiries can be directed to the corresponding author.
